# Supplementary material for: Adsorption and Release of Rose Bengal on Layer-by-Layer Films of Poly(Vinyl Alcohol) and Poly(Amidoamine) Dendrimers Bearing 4-Carboxyphenylboronic Acid
Source: Polymers (Basel). 2020 Aug 18;12(8):1854. doi: 10.3390/polym12081854 (PMC7465977; doi:10.3390/polym12081854)
Supplement: Supplementary file 1 [file polymers-12-01854-s001.pdf]

polymers

MDPI

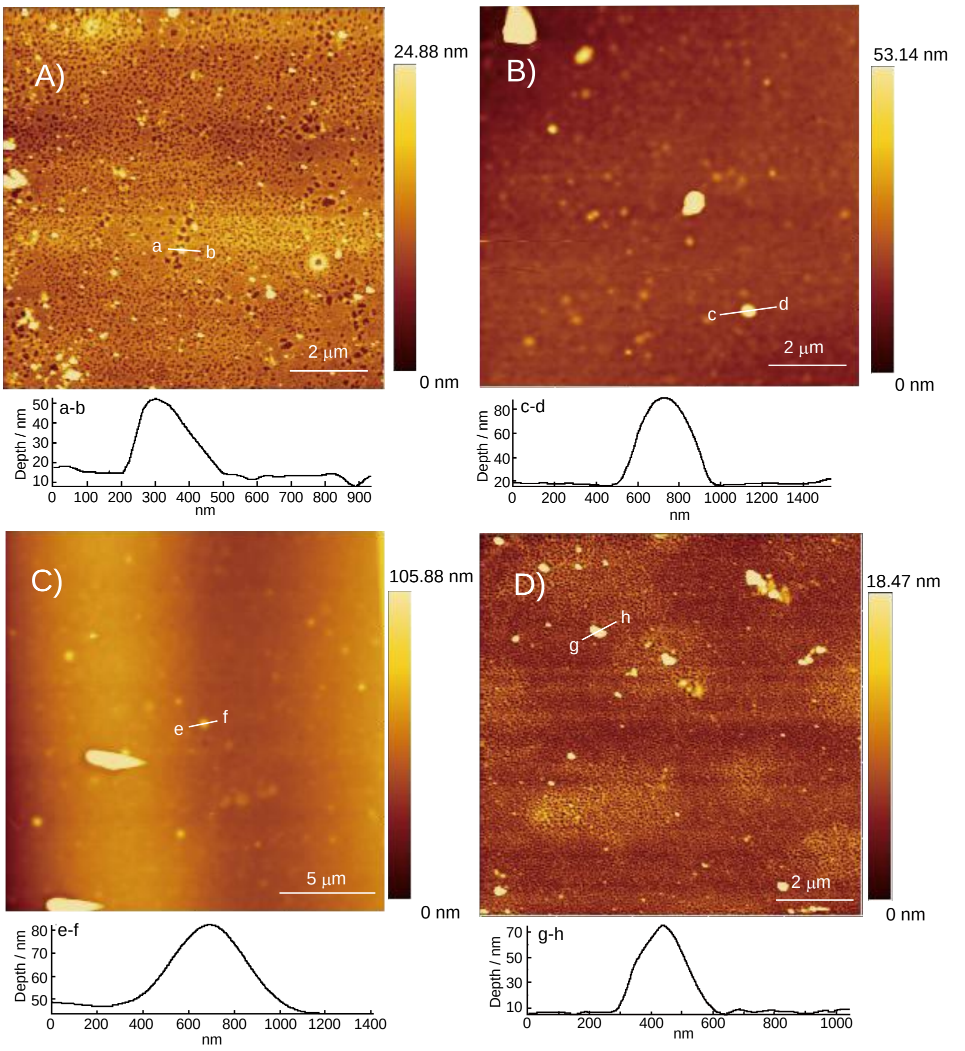

Figure SI1. AFM images and corresponding cross-section profile of (A) one-, (B) three-, (C) five-bilayer-thick (PBA-PAMAM/PVA)$_n$ films. In the AFM image of (D), the (PBA-PAMAM/PVA)$_5$ film was immersed in a 10 mM H_2O_2 solution (pH 7) for 15 min, rinsed with purified water, and dried in a desiccator.

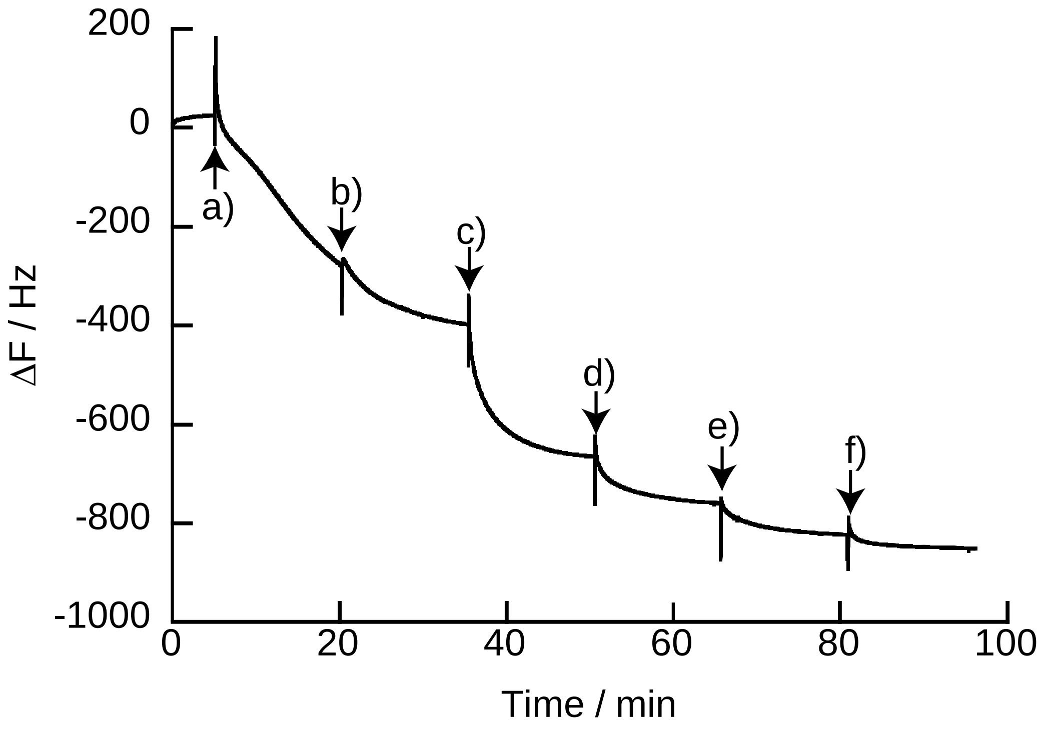

Figure SI2. Frequency change when the (PB-PAMAM/PVA)$_5$ film is immersed in RB solutions (0.1 mg/mL) with various pHs. The LbL films were immersed in RB solutions with (a) pH9, (b) pH 8, (c) pH 7, (d) pH 6 ,(e) pH 5 and (f) pH 6.

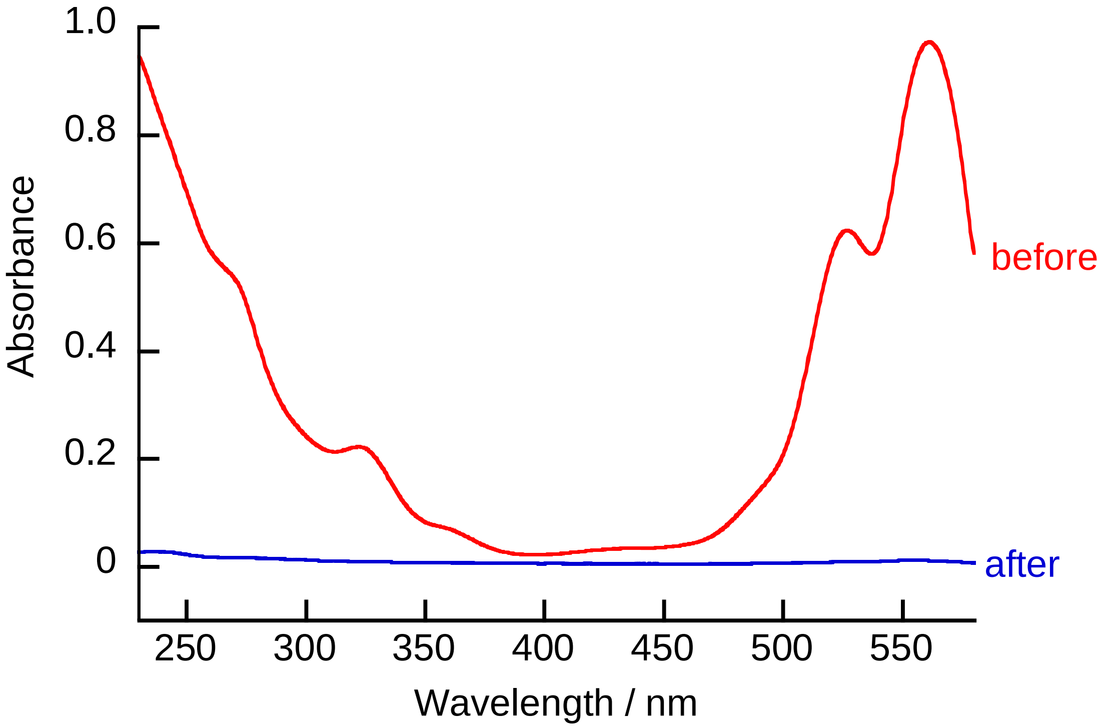

Figure SI3. UV-vis absorption spectra of RB-adsorbed (PBA-PAMAM/PVA)$_5$ film before and after immersion in H_2O_2 solution. The (PBA-PAMAM/PVA)$_5$ film was immersed in RB solution at pH 7 for RB adsorption to the LbL film. The RB-adsorbed (PBA-PAMAM/PVA)$_5$ film was immersed in 100 mM H_2O_2 solution at pH 9.

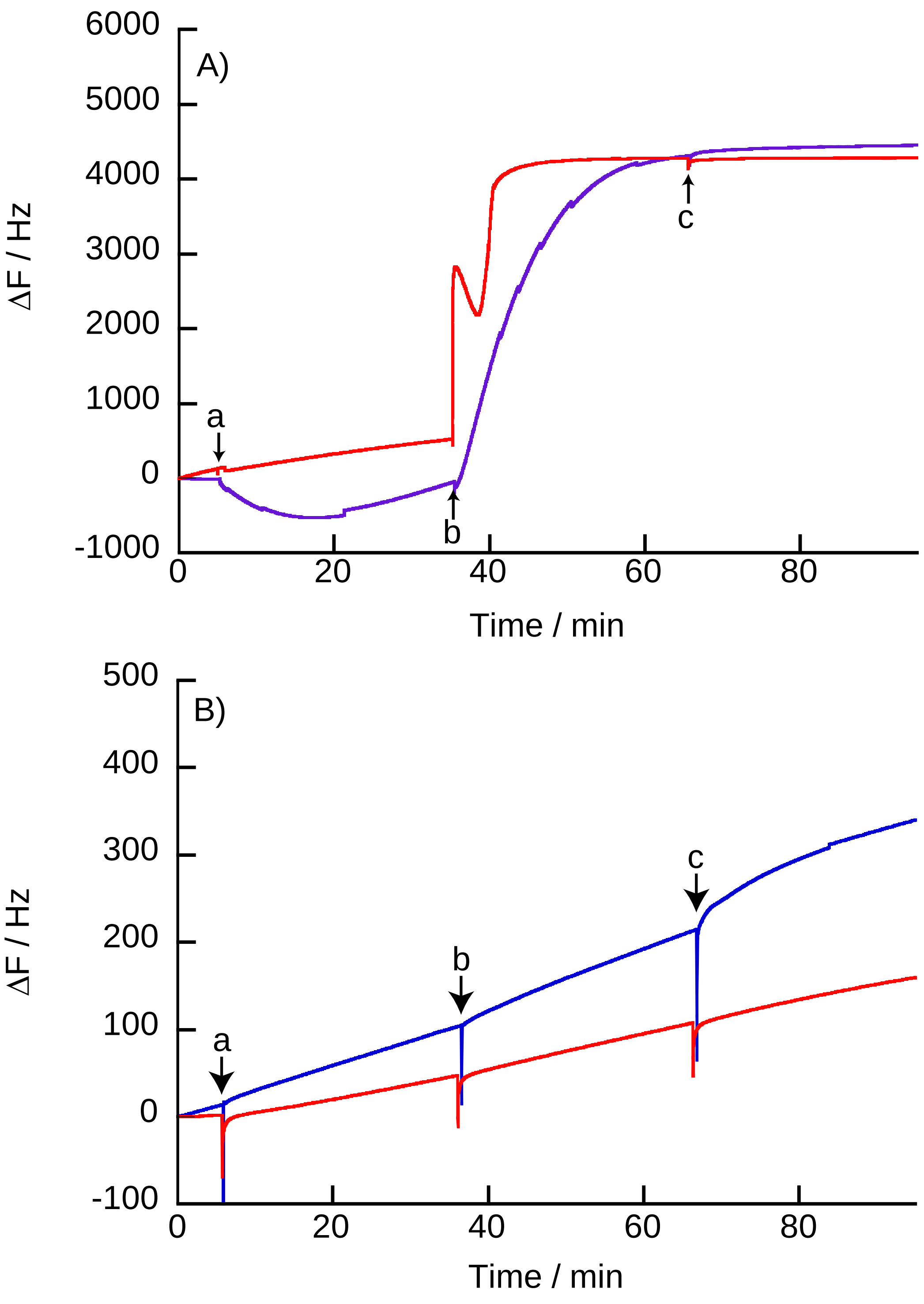

Figure SI4. Frequency change when (A) the (PBA-PAMAM/PVA)$_5$ film and (B) RB adsorbed-(PBA-PAMAM/PVA)$_5$ film are immersed in various H_2O_2 solution at pH 7 (blue line) and pH 4 (red line). H_2O_2 solutions was working buffer containing (a) 1, (b) 10,and (c) 100 mM H_2O_2.

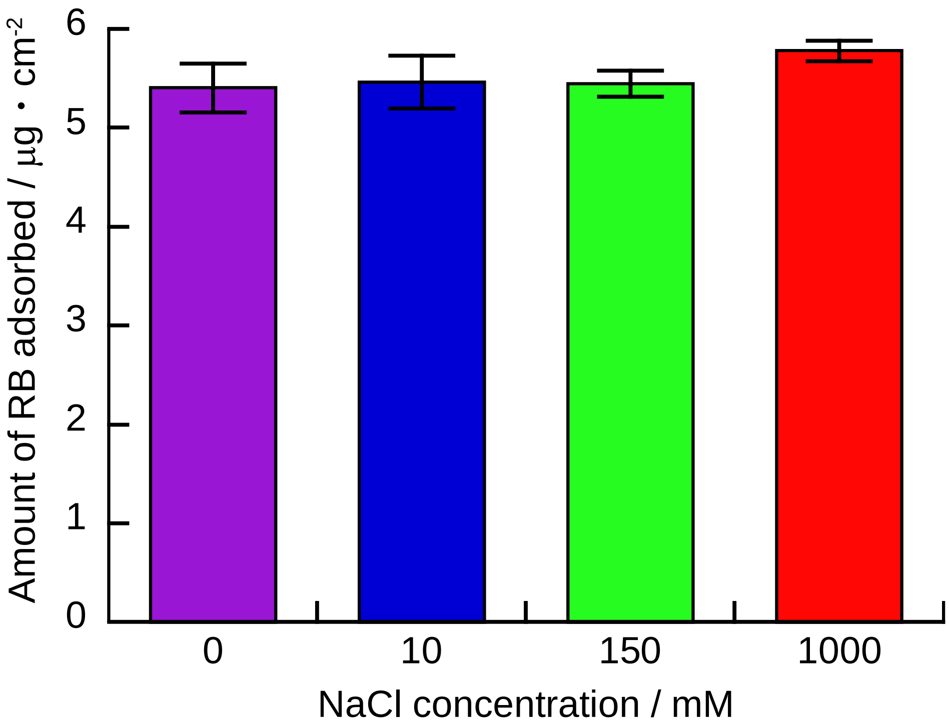

Figure SI5. Amount of RB absorbed on (PBA-PAMAM/PVA)$_5$ films after immersion in RB solution with various NaCl consentrations.

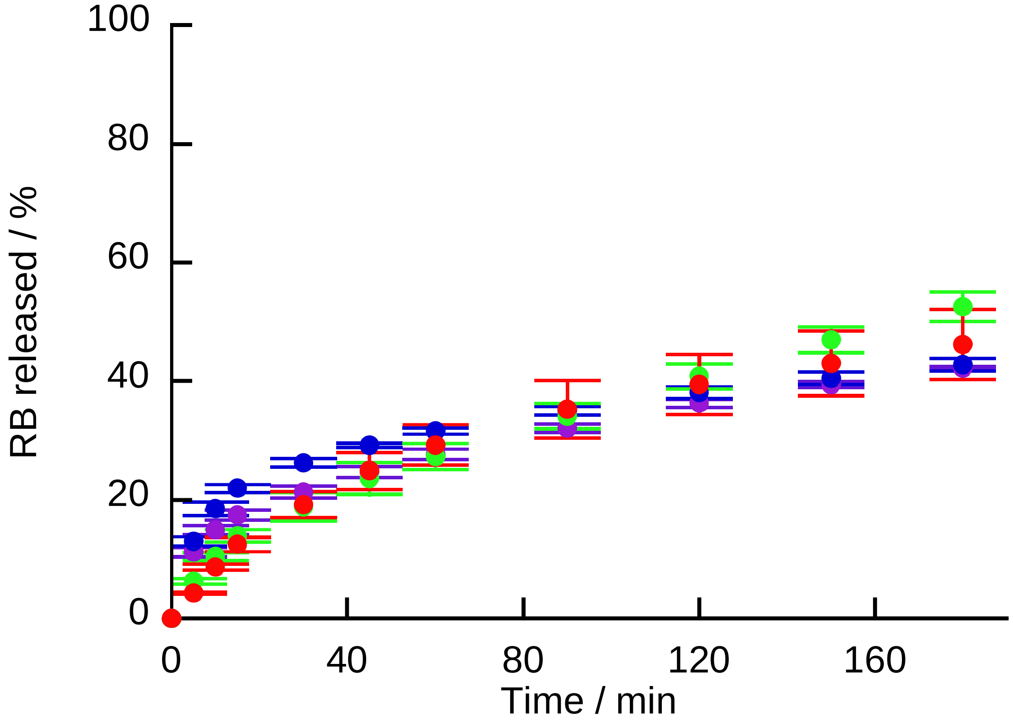

Figure SI6. RB released from (PBA-PAMAM/PVA)$_5$ films immersed in buffers with various NaCl consentrations. The H_2O_2 solutions were working buffer (pH 7) with 0 (purple), 1 (blue), 10 (green), and 100 mM (red) NaCl.
